# The Importance of Liming with an Appropriate Liming Material: Long-Term Experience with a Typic Palexerult

**DOI:** 10.3390/plants10122605

**Published:** 2021-11-27

**Authors:** Miguel Ángel Olego, Miguel Javier Quiroga, Roberto López, Enrique Garzón-Jimeno

**Affiliations:** 1 Research Institute of Vine and Wine, Universidad de León, Avenida de Portugal, 41, CP 24071 León, Spain; germqm@unileon.es (M.J.Q.); jegarj@unileon.es (E.G.-J.); 2 Physical Chemistry Area, Department of Chemistry and Physics, Faculty of Biology and Environmental Sciences, Campus de Vegazana, Universidad de León, CP 24071 León, Spain; rlopg@unileon.es

**Keywords:** aluminium toxicity, dolomitic limestone, long-term, magnesium, rye, soil acidity

## Abstract

Aluminium phytotoxicity is considered the main limiting factor for crop productivity in agricultural acid soils. Liming is a common practice used to improve acidic soil properties, but an appropriate liming material is essential for both agricultural productivity and environmental sustainability. A long-term field experiment with two liming amendments (dolomitic limestone and limestone) was developed during 10 years to determine the changes in soil acidity and assess the effects on crop (rye) yields. Although the adverse effects of the soil acidity conditions were alleviated with both amendments tested, dolomitic limestone was the most effective in the short- and long-term period. In terms of the saturation of exchange complex, dolomitic limestone had a better efficiency, likely based on its rate of dissolution. No significant changes in soil organic matter and exchangeable potassium levels between the treatments tested were found. Both liming materials significantly increased the rye total biomass, but interestingly, significant correlations were showed between tissue levels of magnesium and biomass production, but not between the latter and calcium. The increases in rye biomass production compared with control soils at the end of the research were the following: dolomitic limestone, 47%, and limestone, 32%. A link between an increase in magnesium bioavailability and biomass production was found, as well as between magnesium rye content and total, spike and stem biomass. Hence, it could conceivably be hypothesized that since magnesium is crucial for the transport of assimilates from source leaves to sink organs, alleviating its deficiency leads to avoiding the reducing growth rate of sink organs. Although further investigations are needed to gain a better understanding of liming on the biological, chemical and physical soil properties in the long term, our research provides support for the conceptual premise that an appropriate selection of liming material is crucial for the productivity of acid soils.

## 1. Introduction

Soil acidity naturally develops because of different factors of soil formation acting alone or in combination: parent materials low in bases, and climates favouring strong leaching [1]. Natural soil acidification is favoured in areas of high rainfall, which facilitates the leaching of exchangeable calcium (Ca) and magnesium (Mg) and exhibit a high saturation of aluminium (Al), as it becomes the dominant cation in the exchange complex [2]. However, soil management practices, such as regular N fertilizer application, can also acidify soils; specifically, fertilizers that produce ammonium [3]. Aluminium phytotoxicity is considered the main limiting factor for plant growth on acid soils, trivalent Al being the major cation in the exchange complex when the pH is below 5.5 [2]. Having a similar hydrated ionic radius, Al competes with Mg ions for apoplastic binding sites and plasma membrane Mg transporters [4].

Since liming is mainly practiced to raise the soil pH, most studies have focused on plant productivity, amelioration of soil acidification, Al phytotoxicity and exchangeable cations [5]. In order to overcome previous crop limitations as limitations of Al phytotoxicity, Ca amendments are an agronomic practice commonly used to reduce acidity and Al toxicity in acid soils [6]. The liming material selection in traditional agronomy management, to correct soil acidity and to improve agronomic soil productivity, must be based on several issues in addition to their neutralizing values or calcium carbonate equivalent (CCE). Thus, other constraints, such as mineral nutrient deficiencies associated with soil acidity (availability of the macronutrients Ca, Mg and potassium (K), as well as the micronutrients molybdenum (Mo) and boron (B) that are curtailed in acid soils [7]), must be taken into account.

Magnesium is one of the nine essential macronutrients that is used in large quantities by plants, performing several physiological functions in plant cells: the key atom of chlorophyll, where it acts in pigment–protein complexes to gather photons in both photosystem I and II; involved in CO_2_ assimilation reactions in the chloroplast; as a cofactor and allosteric modulator for more than 300 enzymes; and also involved in photophosphorylation and photoassimilates partitioning and utilization [8]. Its general physiological benefits for active growth often obscure specific mechanisms involved in resistance to disease, being also an important contributor to overall plant health [9]. Despite its abundance in the environment, Mg deficiency often occurs in the field because of soil acidity [10]. Adequate Mg nutrition of crop plants is important for better nitrogen-use efficiency and grain N accumulation, as well as to avoid both of the key physiological processes in plant cells, and impairments in the growth and development of sink organs, being adversely affected [11]. Surprisingly, although a positive impact of Mg on the grain yield of field-grown cereals frequently has been reported, few studies on the significance of Mg on both harvest quality [12] and the mineral composition of tissues of cereals grown under field conditions have been published. Despite Mg deficiency in crops being a common nutritional disorder, particularly in acidic soils, the effects of its nutritional constraints remain little examined compared with other essential elements [13].

Lower soil Mg availability appears under some conditions such as in acidic soils with low cation exchange capacity [14]. Indeed, due to its potential for leaching in highly weathered soils and the interaction with Al, Mg deficiency is a critical concern in acidic soils [15]. Thus, an important consideration in the purchase of liming materials is the type of lime needed based on the soil nutritional constraints. In this regard, liming with Mg amendment sources may be more efficient than liming with Ca amendments under certain soil acidity conditions with low soil Mg availability. In this regard, Ca–Mg liming can represent a valuable alternative to counteract acidification and base cation depletion in acidified agrosystems [16]. Dolomitic limestone comprises mainly the mineral dolomite, which is made of a calcium and magnesium double carbonate (CaMg(CO_3_)_2_). It has two important characteristics as a liming material: (i) its high neutralizing capacity; and (ii) its low dissolution rate [1]. Thus, dolomitic limestone can be a good liming material because of the benefit of simultaneously providing Ca and Mg during the process of counteracting soil acidity [17], especially in those acid soils where soil Mg availability must be improved.

Although limestone is currently the amendment most frequently used to ameliorate soil acidity in the region where the current research was conducted, other liming materials could efficiently replace this amendment because of its better fit to particular nutritional constraints. Therefore, the main aim of this research was to assess the long-term effects (10 years) of one Ca–Mg and one Ca-based liming amendment (namely, dolomitic limestone and limestone) on several soil fertility properties and rye biomass production in an acid soil, with a close inspection of their temporal changes. Additionally, calcium, magnesium and potassium concentrations in rye stem biomass were also studied. This could improve agronomic strategies regarding acid soil management, reflecting the importance of assuming that the presence of Mg in liming materials is mandatory in Mg-deficient situations. The hypothesis that was be tested in this work was that an increase in magnesium bioavailability in acid soils has significant effects on both biomass production and composition, irrespective of a simultaneous increase in calcium bioavailability. It is hoped that this research will contribute to a deeper understanding of liming on acidic soil fertility conditions in the long term.

## 2. Results

### 2.1. Initial Soil Characterisation before Liming

The initial soil characterisation was already reported in our previous work. Therefore, it can be consulted in our previous publication [2].

### 2.2. Temporal Evolution of Soil Parameters

The results obtained from the mixed ANOVAs are reported in Table 1. Based on the results here presented, the significant effect of T on all the properties of the soil studied (pH, Ca, Mg and Al) can be highlighted. However, this was not observed in SOM and K. The effect of T significantly changed with D in the following soil properties: pH, Ca, Mg and Al. Furthermore, D and Y presented a significant effect on T in all the soil properties studied, with the exception of K, as it is determined by the (T × D × Y) significant interaction. With the aim to evaluate where the significant differences between the T means are, a further comparison between all possible variable pairs was assessed by post hoc methods (see Table S1), for both D and Y individually (in spite of the non-significant interaction of T × D × Y already commented for K).

**Figure 1:**
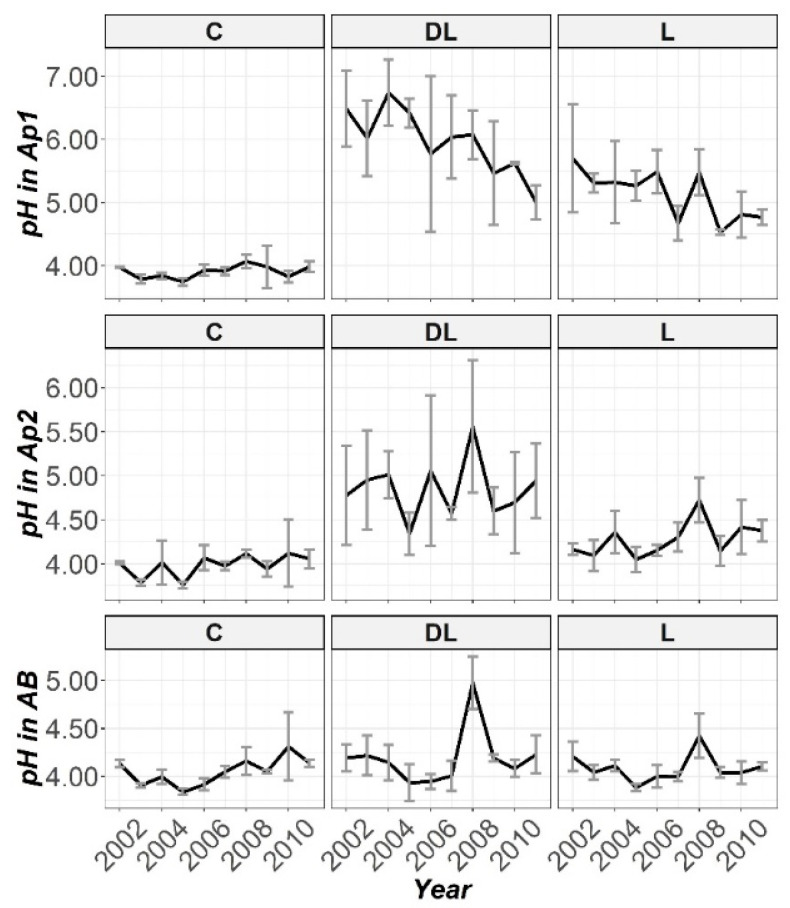
Temporal evolution of pH in the three horizons (Ap1, Ap2 and AB) studied throughout the ten years of soil monitoring (2002–2011).

The temporal trend in pH in all the sample depths studied (Ap1, Ap2 and AB) is displayed in Figure 1. From the data here reported, as a result, DL was found to be the most effective liming treatment to increase the pH of the soil. On closer inspection, at the Ap1 depth, the DL liming treatment increased the pH soil pH by ∽2.0 units during the first four years when compared with the unlimed control, although L produced an increase in the pH of the soil by ∽ 1.5. Liming with both DL and L significantly increased soil pH from the beginning to the end of the research (Table S1; only in year 2009 no significant differences were found between C and L for the soil pH parameter).

**Figure 2:**
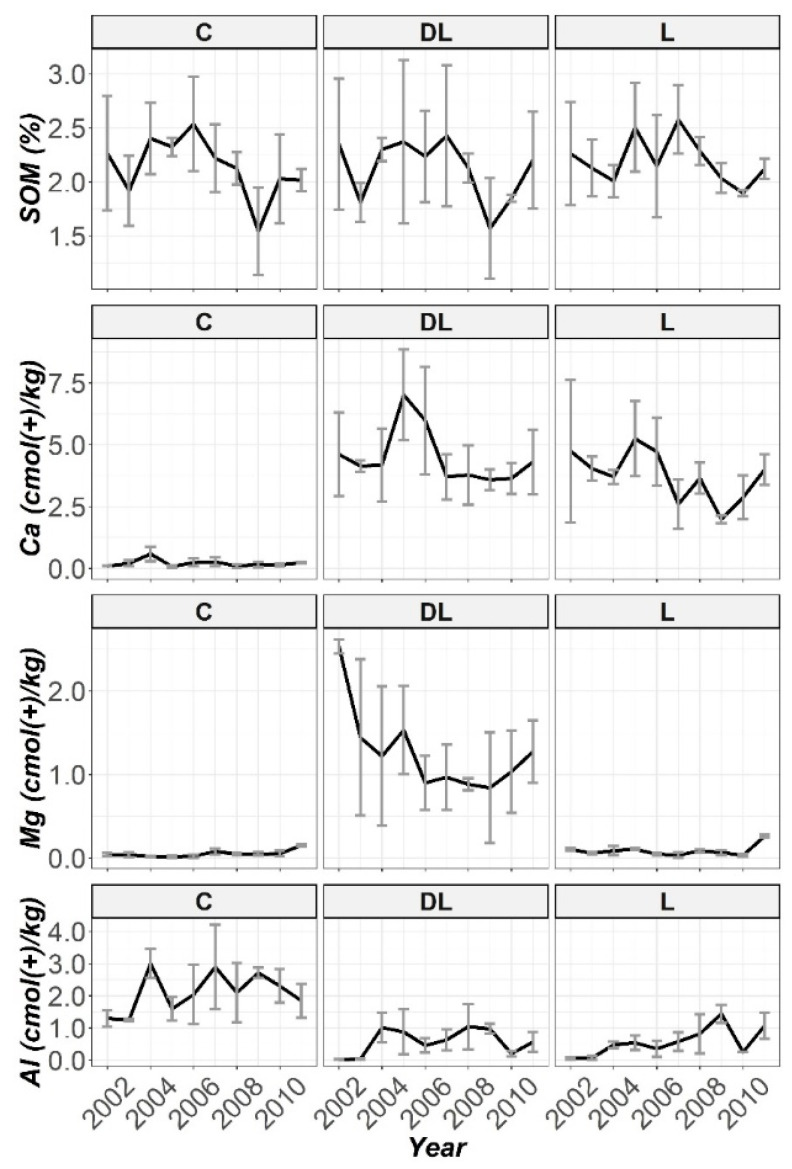
Temporal evolution of SOM, Ca, Mg and Al in the Ap1 horizon throughout the ten years of soil monitoring (2002–2011).

**Figure 3:**
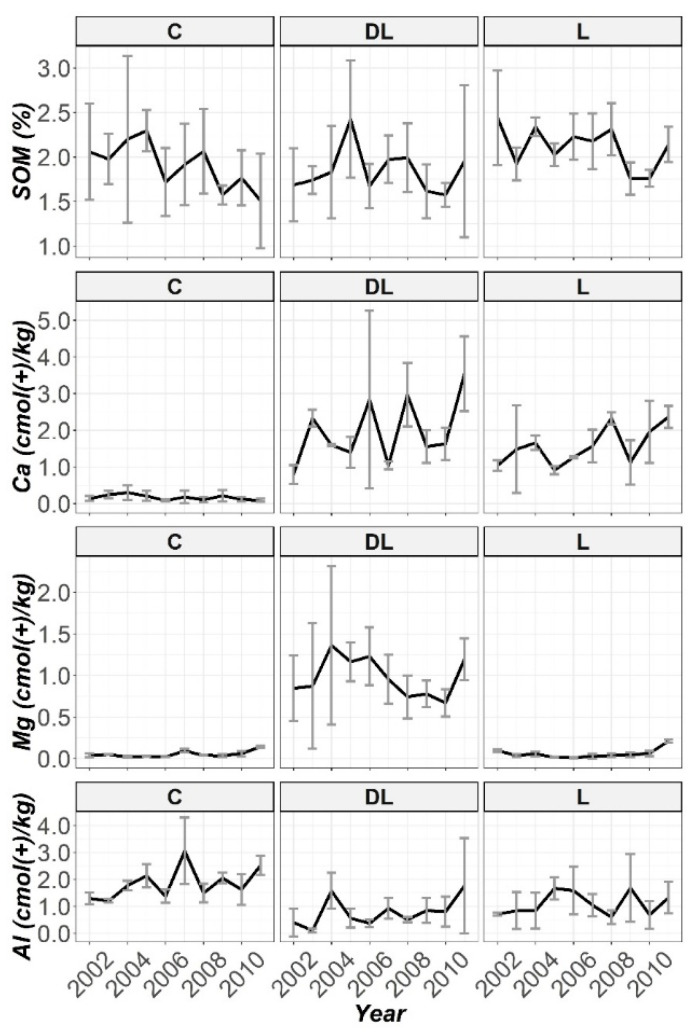
Temporal evolution of SOM, Ca, Mg and Al in the Ap2 horizon throughout the ten years of soil monitoring (2002–2011).

**Figure 4:**
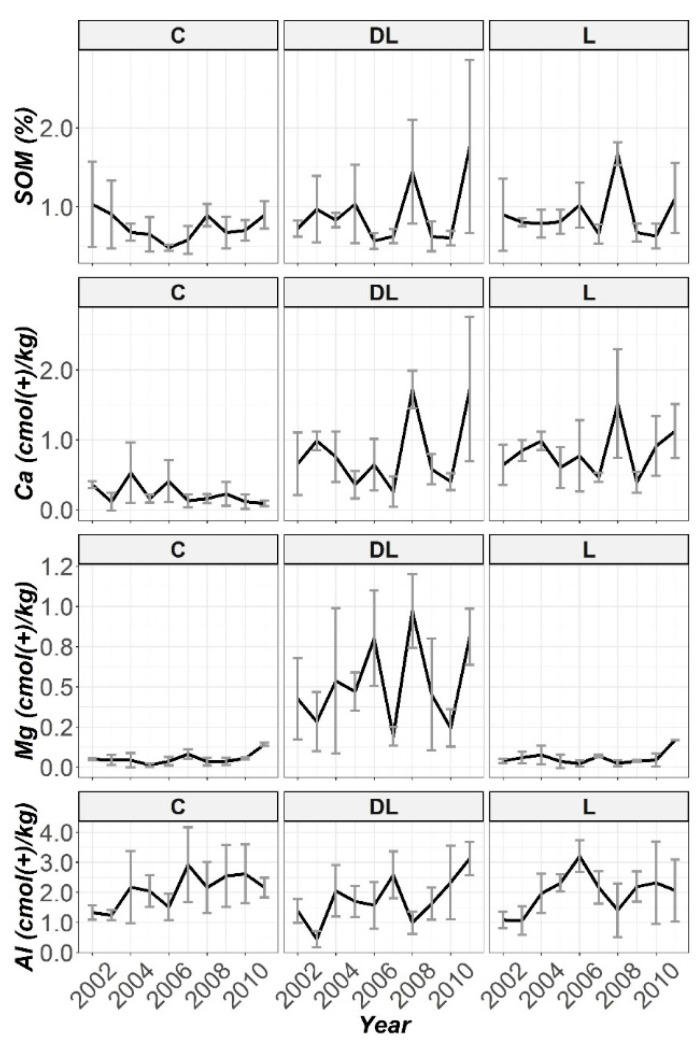
Temporal evolution of SOM, Ca, Mg and Al in the AB horizon throughout the ten years of soil monitoring (2002–2011).

Furthermore, the pattern observed with time of SOM and exchangeable content of Ca, Mg and Al throughout the ten years of monitoring by using the liming treatments and control subplots in all the work depths are subsequently displayed in Figure 2, Figure 3 and Figure 4. No significant differences were found; in practical terms, for the SOM and K soil levels in any of the studied horizons between treatments throughout the research period (Table S1). From these figures, apparently, during the first five years it can be seen that DL showed better efficiency than L in increasing the soil Ca bioavailability in the Ap1 horizon, while in the remaining years (2007–2001) the two liming treatments showed a similar effect, although both treatments significantly increased the Ca levels throughout the research (Table S1). The magnitude of the difference in the Ca levels between limed with DL and the C treatments increased most notably during the years 2005 and 2006, when exchangeable Ca at the Ap1 depth was 100 and 25 times higher, respectively, when compared with the untreated plots. In this sense, at this period of the research the Ca levels in L were higher than C by 75 and 20 times, respectively. At the end of the research (2011), the Ca levels in DL and L were 19 and 17 times higher than C, respectively. Exchangeable Al in this soil layer decreased dramatically in response to liming in 2002 for both DL and L (Al levels in C were 43 and 26 times higher than DL and L, respectively), and this reduction was maintained throughout the research. On the other hand, DL showed significant differences with both C and L subplots in Mg levels throughout the research.

The increase in pH of Ap2 was related to the presence of DL (only liming with DL significantly increased the soil pH levels from the beginning of the research; Table S1). On closer inspection (Figure 1), it can be observed that this increasing trend was maintained during the research period. In this regard, the in-depth lime effect of the liming treatments was observed from the first year in the case of DL treatment for the Ap2 horizon, while in the case of L treatment this effect was shown in a more progressive way. On the other hand, L showed significant differences with C subplots in soil pH levels in years 2005 and 2007. Interestingly, except for the year 2006, at this study depth both liming materials significantly increased the Ca levels from the beginning to the end of the research, whereas only DL showed significant increases in Mg levels throughout the research (Table S1). Exchangeable Mg at this depth was about 68 and 10 times higher when compared with the untreated plots throughout the research, and 8.5 times higher at the end of the research. On the other hand, exchangeable Al at this soil depth significantly decreased in response to liming with both DL and L from 2002 (Table S1), with this reduction pattern through 2011 best sustained by DL treatment.

Finally, at depth AB a marked increase in soil pH was only observed in the seventh year (2008), for both DL and L (only liming with DL significantly increased soil pH in years 2003, 2008 and 2009; Table S1). While DL was fairly more effective than L in increasing soil Ca bioavailability at the Ap2 depth, at this depth both liming treatments showed a similar efficiency (both DL and L significantly increased the soil Ca levels in years 2003, 2008 and 2011, whereas significant differences in this nutrient between L and C were showed in years 2005, 2007 and 2010, while between DL and C only in the year 2009; Table S1). From the charts (Figure 2, Figure 3 and Figure 4) it can be seen that by far the most effective treatment to increase Mg bioavailability in all studied soil horizons was the amendment DL (as was observed in both Ap1 and Ap2, DL showed significant increases in Mg levels throughout the research compared to the C and L subplots; Table S1), whereas both amendments (DL and L) appeared to have a similar efficiency in decreasing the Al concentrations at all studied depths (although exchangeable Al only significantly decreased in response to liming with DL in years 2003 and 2008; Table S1).

### 2.3. Temporal Evolution of Biomass

In agreement with the mixed ANOVA results reported in Table 2, a significant effect on all the biomass parameters by T was found (spike, stem and total biomass, and also Ca and Mg content in stems), barring K-Rye. Furthermore, the effect of Y on the biomass parameters individually was found to be significant. Indeed, Y presented a remarkable effect on T, in consonance with the significant interaction between both factors (T × Y) (see Table 2). For the sake of identifying where the significant differences between T are, a more specific analysis by comparing between all the parameter pairs was performed through post hoc methods (biomass data and cation content in stems data presented in Tables S2 and S3, respectively), but for Y individually (because the significant interaction found in T × Y).

**Figure 5:**
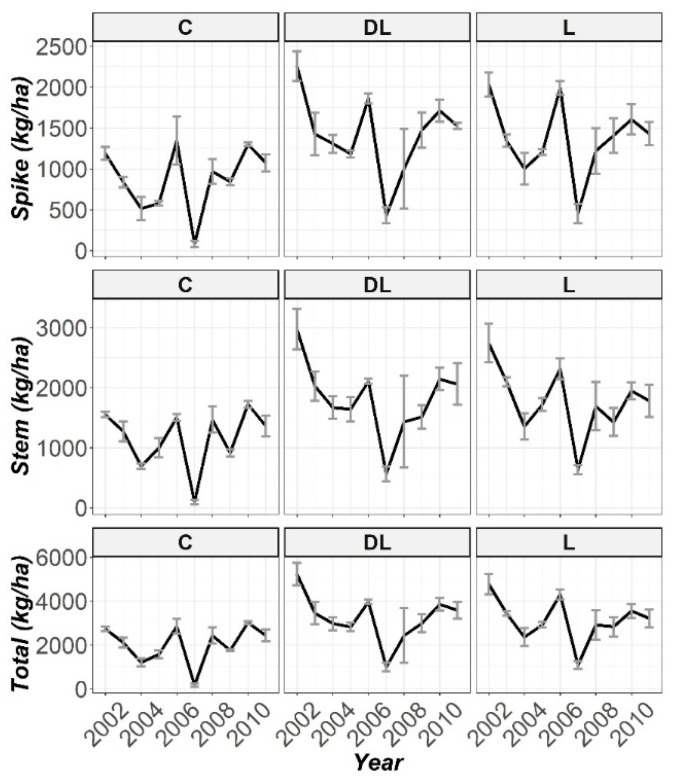
Temporal evolution of the spike, stem and total biomass throughout the ten years of biomass monitoring (2002–2011).

Following the same procedure as for the soil parameters, the temporal evolution throughout the ten years of monitoring of the spike, stem and total rye biomass are displayed in Figure 5. Viewing this figure, a marked increase in the first four years was found for total biomass, and in both spike and stem biomass in the experimental cases where DL was used in liming when comparing with the controls. These results were also observed in those cases where liming happened with L. Interestingly, the biomass levels (all total, spike and stem biomass) were consistently significantly higher throughout the research in DL and L compared to C (Table S2). Only in the year 2004 there was a significant difference in all the rye biomass parameters between the DL and L treatments (DL > L), whereas at the beginning of the research (2002), that significant difference between DL and L only was showed in the spike data (Table S2). At the end of the research, DL and L increased in relation to the total production of rye biomass by 47% and 32%, respectively, as compared to the control soils.

Along 2007, a high decrease in biomass production was observed. The most remarkable reasons of this trend could be the low rainfall and the low average annual temperature for that year in the research area. These hypotheses are based on the climate characteristics of the area, which presents a yearly average temperature of 9.4 °C and a yearly average precipitation of 600–850 mm [18]. Curiously, in a closer observation the buffer effect of DL and L compared with C on biomass production in this year can be highlighted. Specifically, the total biomass levels in DL and L were approximately 6 times higher than C in this year.

**Figure 6:**
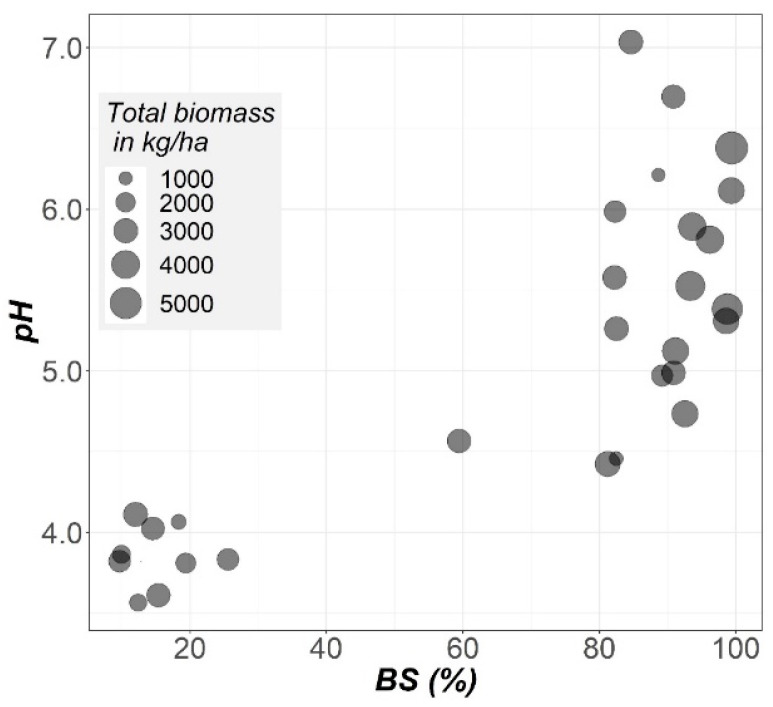
Relationship between soil pH, base saturation of the effective cation exchange capacity (BS) and total rye biomass.

The relationship between the total biomass, soil pH and base saturation in Ap1 (BS; calculated as the percentage of effective cationic exchange capacity occupied by base cations (Ca, Mg and K)) is shown in Figure 6. Viewing this figure, it can be observed that the higher total biomass production is mainly presented in the area of the graph belonging to both greater BS and soil pH.

**Figure 7:**
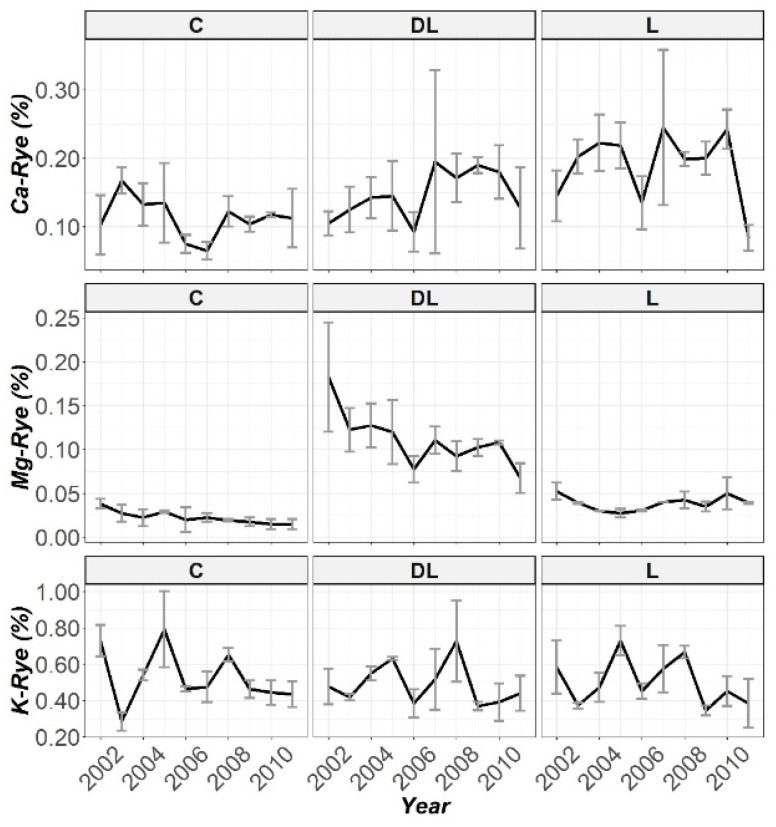
Temporal evolution of the Ca, Mg and K levels in stems throughout the ten years of cation content during stem monitoring (2002–2011).

The temporal progression of the Ca-Rye, Mg-Rye and K-Rye levels are displayed in Figure 7. Specifically, from the beginning until the end of the research, a significant and prolonged increase in Mg-Rye between DL and both the L and C subplots was observed. On the other hand, significant increases in Ca-Rye concentrations with regard to the C subplots were observed from the second year of the research (2003) until one year before the end of the experiment (2010), almost exclusively by the L treatment. Finally, none of the liming materials seem to have modulated any particular trend in the K concentration in rye stems (Table S3).

### 2.4. Correlations between Soil and Biomass Parameters

Relationships between the soil parameters at the Ap1 horizon, as well as between the biomass production and nutrient (Ca, Mg and K) tissue levels were investigated to evaluate potential links (Table 3). There were several strong overall relationships (Pearson correlation ≥ ±0.50) between the soil and biomass parameters. Among these, special mention should be made of the following: pH and Mg-Rye, Ca and Mg-Rye, Mg and Mg-Rye, Al and Spike, Al and Stems as well as Al and Total. Specifically, those linked to biomass production and Mg content in biomass tissues were moderates (Pearson correlation ≤ ±0.49–±0.30), while those linked to SOM and K were weak (Pearson correlation < ±0.30).

## 3. Discussion

**Figure 8:**
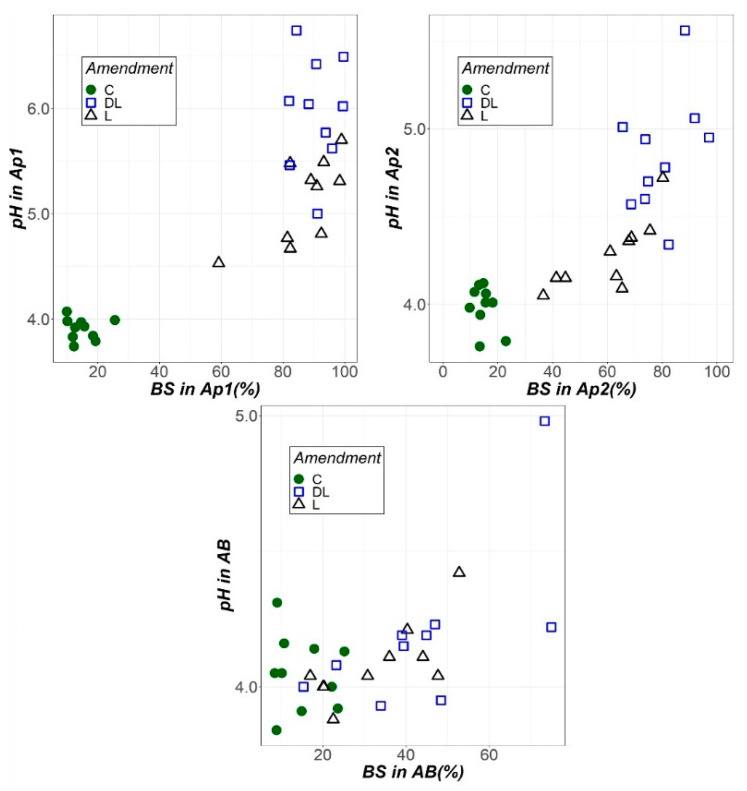
Soil pH and base saturation (BS) as a function of the liming treatment (control (C), dolomitic limestone (DL) and limestone (L)).

As a general comment, the soil liming with DL and L improves the soil chemical characteristics, in agreement with the well-established literature on the matter [19,20]. From the results reported in this manuscript, liming with DL could be considered the most interesting treatment concerning plant nutrition. The bioavailability of plant nutrients depends on the soil pH and, therefore, pH affects the crop plant growth [18]. In this sense, DL is presenting as the most interesting treatment to ameliorate the pH. In order to highlight this result, Figure 8 is presented in terms of soil pH and the base saturation levels (the latter represented as the percentage of ECEC occupied by soil bases (Ca, Mg and K)), for the three soil horizons studied [2] (Ap1, Ap2 and AB). As can be seen, DL was more effective than L in increasing the soil pH in both Ap1 and Ap2, whereas both amendments had a similar behaviour in the AB horizon. In terms of BS, DL seems to have a slightly better efficiency than L in all the soil horizons monitored. As we found in our previous work [2], DL and L reactivity (in terms of dissolution), which is also dependent on the hardness and the particles size [18], can be behind this result since the ability of the soil pH increasing and also the base saturation levels are strongly related with DL and L reactivity. It is also true that the higher CCE of DL (see Table 3 in [2]) cannot be neglected.

Because amendments were incorporated immediately after their application, both liming materials increase markedly the soil pH immediately rather than gradually over time (especially in the top horizon (Ap1), when the soil acidity was reduced very significantly). This is in line with other studies [20] reflecting that the way in which the liming materials were applied in the field influences the effect on soil pH (surface application, not incorporated into the soil through mobilization, does not increase much the soil pH immediately). On the other hand, although liming materials application can be slow or, in some cases, even ineffective in increasing subsoil pH [21], in this research this increase was observed from the beginning in the Ap2 horizon, as well as in 2008 and 2009 in the AB horizon for both liming materials. The above indicates that the downward movement of exchangeable Ca and Mg occurs after exchangeable sites in the Ap2 horizon were saturated by both ions. However, this impact on subsoil pH was lower in L than in the DL subplots, and the higher efficiency of DL over L in decreasing the subsoil acidity was evident immediately after the amendments were applied.

Consistent with the literature [22,23,24,25], the overall effect of the liming treatments on the soil Al levels was a decrease in its effective Al saturation on the exchange complex, as well as an increase in the availability of Ca and Mg for all the horizons studied. As expected, Al was efficiently reduced with both liming materials, but interestingly both Ca and Mg bioavailability was markedly increased in those subplots limed with DL. It can then be expected that the lower solubility of the L amendment relative to DL might account for its lesser effect on both pH and exchangeable Ca in the Ap1 and Ap2 horizons. In addition to the above, because both Al and Mg ions have a remarkably similar hydrated radius, causing the Mg uptake system or the Mg-binding sites on enzymes to not distinguish well between both ions [4], it is appropriate to suggest that DL was a better option than L as a liming material in the Ca- and Mg-deficient soils in this research. Moreover, taking into account the theoretical low dissolution rate of DL [1], it is very likely that an LR calcium-based calculated, as in this research, may be a better strategy than an LR CCE-based one calculated in those acid soils with very low exchangeable Mg.

Contrary to expectations, although soil amendment with liming materials can accelerate the mineralization of SOM through increasing the soil pH, reducing the SOM content in the soil [26], the observed differences in SOM levels in the soil profile between treatments in this research were not significant. These results are in agreement with those of Crusciol et al. (2017) [27], who suggested that the change in SOM content may take longer or the degree of change in soil pH could not be sufficient to change the SOM levels. However, these results are in disagreement with those obtained by Chan and Heenan [28], who presented a reduction in the SOM content after increasing the pH of the soil and a concomitant enhancement in microbial activity. On the other hand, because liming produces a more favourable environment in the soil to improve the plant growth, it is expected that the plant productivity increases after liming, thus presenting higher organic matter inputs [29]. Despite the above, it is important to bear in mind the size of the liming materials, as DL and L are key factors in regulating soil organic carbon mineralization in acidic soils when those amendments are applied to manipulate its chemical attributes [17]. Thus, because several reports have shown both an increase and decrease in SOM after liming [30,31,32,33], these data must be interpreted with caution, and more research on this topic needs to be undertaken before the association between liming and SOM evolution is more clearly understood.

Another important aspect to be discussed is the addition of high levels of Ca and Mg in the soil. If these levels are over those that are suitable to the ion-exchange operation, the selectivity of Ca and Mg for these sites produces a displacement of K from the exchange complex, thus increasing the potential for loss by leaching [34]. Thus, although it could be expected that liming decreased the exchangeable K, this effect was not significant after liming. With respect to the above, although DL is recommended for soils deficient in Mg, using it too frequently can result in Mg indices > 3, and so poor K availability [18]. Indeed, it should be noted that despite the beneficial effects of liming over soil acidity, inadequate liming rates, i.e., overliming, could create deficiencies in macronutrients and micronutrients [35]. Particularly, overliming with dolomitic limestone could result in higher Mg/K rates and so poor K availability [18]. Thus, an appropriate fertilization scheme to accompany liming could be mandatory.

In our research, liming with both DL and L significantly increased the rye total biomass from the beginning of the research (as well as spike and stem biomasses), maintaining this effect during the whole experiment. According to these data, we can infer that biomass production improvement might be attributed to the enhancement in the rye growth environment that resulted from the increase in soil pH, the reduction in Al levels and the supply of Ca and Mg. However, for the duration of this research, DL maintained the soil pH levels in the Ap1 horizon above values below which rye growth may be restricted on mineral soils (4.90; [18]), while for L from 2002 to 2006, and in 2008. In this sense, during the first three years of the research (2002–2004), biomass production in the DL subplots was higher than in the L ones (being some of these differences between the two liming treatments significant). This range of years corresponded to the period when the available soil Mg levels in the DL subplots were very clearly higher. These results are suggestive of a link between an increase of Mg bioavailability and biomass production. One of the findings that emerges from these data is that at low soil pH (with severe constraints on both soil Ca and Mg bioavailability), for acid-sensitive crops such as rye, there will be greater benefits from both liming materials, as the liming material is a source of Ca and Mg, rather than liming with an exclusively Ca-based liming material. Furthermore, Mg could be displaced by the large amounts of Ca added when liming exclusively with L. Accordingly, the exchangeable Mg content might decrease in the topsoil of the L-treated plots, and increase through leaching into the subsoil horizons [36]. Thus, although liming improves the soil conditions for plant growth, the addition of large amounts of Ca could result in lower Mg availability to plant roots [14]. The above could lead to limited rye growth, most probably by downregulation of photosynthesis activity with sugar accumulation in the source leaves as a major consequence of a Mg shortage [13]. Furthermore, irrespective of the liming material used, inappropriate liming rates may result in a reduction in the availability not only of those commented (K and Mg) but also other nutrients such as manganese [25,37] and phosphorus [38], with a negative impact on crop production.

Soil correction with both amendments efficiently raised the Ca and Mg levels in rye tissues. Particularly, DL and L increased the Ca concentration in rye shoots more markedly during the period 2007–2010, whereas only DL increased the Mg concentration in rye tissues (interestingly, from the beginning of the research); we did not find any effect of lime amendments on the K-rye levels. Although, Kinraide and Parker (1987) [39] showed that Mg was found to have less effect than Ca on ameliorating Al toxicity in wheat, Souza et al. (2006) [40] observed that soil correction increased the base saturation (Figure 8) and, consequently, the Ca and Mg levels in the dry matter of crop tissues. Indeed, significant correlations between Mg-Rye and total, spike and stem biomass (Table 3) are consistent with the above. These results are in agreement with the ideas of Senbayram et al. (2015) [41], who remarked that the effects of crops’ Mg nutrition on photosynthesis and transport of photosynthates, as well as its influence in enhancing nutrient utilization, and are consistent with those of Tan et al. (1993) [42], who reflected that high Mg levels in a solution reduced sorghum sensitivity to Al and at high rates increased the dry matter yield. The above accords with earlier observations that showed how plants with an increased Mg uptake and content in the cytosol are resistant to Al toxicity [43]. Hence, it could conceivably be hypothesized that since Mg is key for the transport of assimilates from source leaves to sink organs, the resulting Mg-deficiency stress increasing the assimilates accumulation in the source leaves, reducing the growth rate of the sink organs [41], an erroneous liming material choice limits both agricultural productivity and environmental sustainability.

## 4. Materials and Methods

### 4.1. Study Site

The soil under study corresponds to an acid Typic Palexerult (USDA, 2010), located in the village of Camposagrado (municipality of Rioseco de Tapia, León, Spain). The research evaluated a *Secale cereale* L. crop (rye) over a period of ten cropping years (2002–2011). Specifically, the long-cycle rye variety used throughout the research was “Ordalie”. The main characteristics have been described in our previous work [2]).

### 4.2. Characterisation of the Liming Materials and Doses

Table 4 shows the mineral composition of the two liming materials used in this study as well as its Calcium Carbonate Equivalent (CCE) [44]. The limestone exhibited the highest calcium oxide content but dolomitic limestone the higher CCE. A liming rate was calculated for the first 35 cm of the soil with the aim of decreasing the Al saturation of the effective cation exchange capacity (ECEC) below 20%, to ensure an adequate degree of base saturation (i.e., 80%), required in general by most annual and permanent crops [45]. The ECEC can be defined as the total amount of exchangeable cations, which are mostly bases, in non-acidic soils, and bases plus aluminium in acidic soils. In such a way, ECEC corresponded to the arithmetic sum of the concentrations of exchangeable calcium, magnesium, potassium and aluminium (the sodium concentrations in the soil under study were negligible).

Rather than the CCE of the liming materials, in order to add the same CaO content, the lime requirement (LR) was calcium-based calculated using the known Cochrane’s formula [46], which takes the levels of exchangeable aluminium, calcium and magnesium in the soil into account as ECEC, and fix a required % aluminium saturation (RAS) of the ECEC (LR (CCE Mg/ha) = f(Aluminium-RAS (ECEC)/100)), where aluminium and ECEC are in cmol kg^−1^ soil and f is a crop factor. This yielded a value of about 6.4 Mg CCE/ha, which corresponds to about 7.7 Mg/ha of limestone (L) and 10.9 Mg/ha of dolomitic limestone (DL). For a complete comparation, a control treatment (C) without applications was used. The liming materials were uniformly spread onto the entire surface of the plots, being incorporated into the soil at a depth of approximately 20 cm using a rotovator pass.

### 4.3. Experimental Design

The experimental design and the statistical analyses were similar to those explained in our previous work on this matter [2]. In this experimental design, liming treatments (T) with three levels (control (C), dolomitic limestone (DL) and limestone (L)), soil depth (D) (with three levels: Ap1, Ap2 and AB as soil horizons) and sampling year (Y) (with ten levels: 2002–2011) constituted the three factors for which significant effects were studied.

### 4.4. Statistical Analyses and Soil and Biomass Analyses

The statistical analyses and soil and biomass analyses were performed following the detailed description included in our previous work [2]. With respect to the biomass analyses, all the measures were performed as dry biomass.

## 5. Conclusions

This study set out to show the importance of selecting an appropriate liming material when soil acidity is conditioned by soil Mg-deficient situations. The results reported showed that liming treatment of these acid soil situations with only calcium-based materials does not provide the best bases:aluminium ratio in the soil exchange complex and rye biomass production. Although successful management of soil acidity depends on both social and economic factors, from a technical point of view it is imperative to optimally select the liming material to reach both profitable agricultural productivity and environmental sustainability.

It is remarkable that the correlation analysis revealed that the magnesium concentration in tissues was positively and significantly correlated with biomass production, but not in the case of calcium. This could reflect that the vital functions that magnesium has in many biochemical and physiological pathways are still constrained when liming is based exclusively on calcium-based liming amendments. Because some of the magnesium-dependent functions can be important in increasing aluminium resistance in plants, it should not be ignored that the strategy for the management of soil acidity should rely on correct diagnosis of the major limitations.

Our research offers some important insights into soil acidity management because this knowledge can be integrated into acidic soil production systems to avoid the acceleration of its degradation, highlighting the need to address this issue through the development of adequate management strategies for these soil types. However, further investigation and experimentation into the long-term effects of lime application on soil properties is strongly recommended to establish a greater degree of accuracy on this matter.

## Figures and Tables

**Table 1:** Analysis of variance performed on soil parameters (pH, SOM, Ca, Mg, K and Al). The variability in the soil parameters was evaluated using the hierarchical multilevel model (maximum likelihood (ML) ratio). The results were significant at * *p* < 0.05 and *** *p* < 0.001.

Soil Parameter	ML Ratio (T)	ML Ratio (D)	ML Ratio (Y)	ML Ratio (T × D)	ML Ratio (T × D × Y)
pH	132 (***)	161 (***)	22.0 (*)	216 (***)	181 (***)
SOM	2.62 (0.27)	404 (***)	35.5 (***)	7.29 (0.12)	131 (***)
Ca	135 (***)	195 (***)	197 (***)	177 (***)	379 (***)
Mg	316 (***)	36.2 (***)	17.0 (*)	79.9 (***)	195 (***)
K	4.58 (0.10)	28.2 (***)	49.5 (***)	1.70 (0.79)	89.4 (0.08)
Al	84.8 (***)	85.6 (***)	33.6 (***)	53.8 (***)	147 (***)

**Table 2:** Analysis of variance performed on the biomass parameters (spike, stem and total biomass; Ca, Mg and K content in stems) at the harvest stage. The variability in the biomass parameters was evaluated using the hierarchical multilevel model (maximum likelihood (ML) ratio). The results were significant at ** *p* < 0.01 and *** *p* < 0.001.

Biomass Parameter	ML Ratio (T)	ML Ratio (Y)	ML Ratio (T × Y)
Spike	112 (***)	50.5 (***)	58.1 (***)
Stem	91.0 (***)	47.6 (***)	59.1 (***)
Total	104 (***)	49.2 (***)	61.5 (***)
Ca-Rye	40.0 (***)	23.8 (**)	45.2 (***)
Mg-Rye	174 (***)	26.6 (**)	67.1 (***)
K-Rye	2.82 (0.24)	38.4 (***)	37.5 (**)

**Table 3:** Pearson correlations between the Ap1 soil (pH, SOM, Ca, Mg, K and Al) and biomass parameters (spike, stem and total; Ca, Mg and K content in stems (Ca-Rye, Mg-Rye and K-Rye, respectively)) throughout the ten years of monitoring (2002–2011) (*n* = 30). The results were significant at * *p* < 0.05, ** *p* < 0.01 and *** *p* < 0.001.

	pH	SOM	Ca	Mg	K	Al	Spike	Stem	Total	Ca-Rye	Mg-Rye	K-Rye
pH	1.00											
SOM	0.09	1.00										
Ca	0.89 (***)	0.15	1.00									
Mg	0.74(***)	0.02	0.57 (***)	1.00								
K	−0.23	−0.02	−0.04	−0.04	1.00							
Al	−0.73 (***)	−0.04	−0.78 (***)	−0.44 (*)	0.11	1.00						
Spike	0.48 (**)	−0.17	0.54 (**)	0.39 (*)	0.07	−0.61 (***)	1.00					
Stem	0.51 (**)	−0.09	0.56 (**)	0.42 (*)	0.07	−0.67 (***)	0.96 (***)	1.00				
Total	0.50 (**)	−0.13	0.55 (**)	0.41 (*)	0.07	−0.65 (***)	0.99 (***)	0.99 (***)	1.00			
Ca-Rye	0.27	0.00	0.30	−0.12	−0.40	−0.49 (**)	−0.07	−0.03	−0.05	1.00		
Mg-Rye	0.83 (***)	−0.01	0.61 (***)	0.95 (***)	−0.18	−0.54 (**)	0.40 (*)	0.41 (*)	0.41 (*)	0.05	1.00	
K-Rye	0.03	0.52 (**)	−0.03	−0.09	−0.12	0.09	−0.28	−0.16	−0.22	0.07	−0.06	1.00

**Table 4:** Chemical composition of the liming materials expressed as dry matter (*n* = 3).

Treatment	CaO ^a^	MgO ^a^	K_2_O ^a^	Al ^b^	CCE ^c^	OM ^c^
Dolomitic limestone (DL)	311	184	3.50	9529	1.01	0.00
Limestone (L)	437	20.8	3.50	7870	0.83	0.00

^a^ Calcium, magnesium and potassium oxide (CaO, MgO and K_2_O respectively) in g/kg; ^b^ Al (Aluminium) in mg/kg; ^c^ CCE (calcium carbonate equivalent) and OM (organic matter) in %.

## Data Availability

Data is contained within the article and Supplementary Materials.

## Supplementary Materials

The following are available online at https://www.mdpi.com/article/10.3390/plants10122605/s1, Table S1: Means and standard deviations (SD) of soil properties pH, SOM (soil organic matter in %), Ca, Mg, K and Al (calcium, magnesi-um, potassium and aluminium soil content respectively, in cmol (+)/kg) during ten years (2002–2011), Table S2: Means and standard deviations (SD) of biomass (Biomass: total rye biomass; Spike: spike rye biomass; Stem: stem rye biomass (all of them in kg/ha)) during ten years (2002–2011). Table S3. Means and standard deviations (SD) of calcium, magnesium and potassium content in rye biomass (Ca-Rye, Mg-Rye and K-Rye respectively; all of them in %) during ten years (2002–2011).
