# Supplementary material for: The Importance of Liming with an Appropriate Liming Material: Long-Term Experience with a Typic Palexerult
Source: Plants (Basel). 2021 Nov 27;10(12):2605. doi: 10.3390/plants10122605 (PMC8707975; doi:10.3390/plants10122605)
Supplement: Supplementary file 1 [file plants-10-02605-s001.zip › plants-1470562-supplementary.pdf]

Article – Supplementary materials

The Importance of Liming with an Appropriate Liming Material: Long-Term Experience with a Typic Palexerult

Miguel Ángel Olego [1,*], Miguel Javier Quiroga [1], Roberto López [2] and Enrique Garzón-Jimeno [1]

[1] Research Institute of Vine and Wine, Universidad de León, Avenida de Portugal, 41, CP 24071 León, Spain; germqm@unileon.es (M.J.Q.); jegarj@unileon.es (E.G.-J.)
[2] Physical Chemistry Area, Department of Chemistry and Physics, Faculty of Biology and Environmental Sciences, Campus de Vegazana, Universidad de León, CP 24071 León, Spain; rlopg@unileon.es
* Correspondence: molem@unileon.es

SUPPLEMENTARY MATERIALS

Table S1. Means and standard deviations (SD) of soil properties pH, SOM (soil organic matter in %), Ca, Mg, K and Al (calcium, magnesium, potassium and aluminium soil content respectively, in cmol (+)/kg) during ten years (2002-2011). Values within each column followed by different lowercase letters are significantly different according to Tukey's honest significance test with Holm-Bonferroni adjustment (0.05). Y: Year of sampling; D: Depth of sampling (Ap1 horizon: 0-12 cm, Ap2 horizon: 12-25 cm and AB horizon: 25-35 cm); T: Liming treatment (C: control; DL: dolomitic limestone; L: limestone).

Y	D	T	pH		SOM		Ca		Mg		K		Al	
			Mean	SD	Mean	SD	Mean	SD	Mean	SD	Mean	SD	Mean	SD
2002	Ap1	C	3.97 a	0.01	2.27 a	0.53	0.10 a	0.01	0.04 a	0.02	0.09 a	0.03	1.30 b	0.26
2002	Ap1	DL	6.49 b	0.60	2.35 a	0.61	4.61 b	1.68	2.53 b	0.09	0.08 a	0.01	0.03 a	0.01
2002	Ap1	L	5.70 b	0.86	2.26 a	0.48	4.74 b	2.88	0.10 a	0.01	0.07 a	0.01	0.05 a	0.03
2002	Ap2	C	4.01 a	0.02	2.06 ab	0.54	0.14 a	0.07	0.04 a	0.03	0.07 ab	0.01	1.29 b	0.22
2002	Ap2	DL	4.78 b	0.57	1.69 a	0.41	0.80 b	0.26	0.85 b	0.39	0.06 a	0.01	0.40 a	0.51
2002	Ap2	L	4.16 a	0.06	2.44 b	0.53	1.04 b	0.14	0.10 a	0.02	0.10 b	0.04	0.71 a	0.06
2002	AB	C	4.13 a	0.04	1.03 a	0.54	0.36 a	0.05	0.05 a	0.01	0.04 a	0.00	1.33 a	0.23
2002	AB	DL	4.19 a	0.14	0.72 a	0.10	0.66 a	0.44	0.43 b	0.25	0.05 a	0.01	1.39 a	0.40
2002	AB	L	4.21 a	0.15	0.90 a	0.45	0.64 a	0.29	0.04 a	0.01	0.05 a	0.01	1.08 a	0.27
2003	Ap1	C	3.79 a	0.07	1.92 a	0.32	0.20 a	0.12	0.04 a	0.03	0.06 a	0.02	1.24 b	0.05
2003	Ap1	DL	6.02 c	0.60	1.81 a	0.18	4.14 b	0.24	1.44 b	0.94	0.07 a	0.01	0.03 a	0.01
2003	Ap1	L	5.31 b	0.15	2.13 a	0.26	4.04 b	0.49	0.06 a	0.02	0.08 a	0.05	0.07 a	0.06
2003	Ap2	C	3.79 a	0.04	1.98 a	0.28	0.24 a	0.10	0.05 a	0.00	0.07 a	0.02	1.20 b	0.07
2003	Ap2	DL	4.95 b	0.57	1.74 a	0.16	2.33 b	0.23	0.87 b	0.75	0.08 a	0.00	0.10 a	0.08
2003	Ap2	L	4.09 a	0.17	1.92 a	0.18	1.48 b	1.19	0.04 a	0.02	0.06 a	0.02	0.84 b	0.68
2003	AB	C	3.91 a	0.03	0.90 a	0.43	0.11 a	0.13	0.05 a	0.03	0.06 a	0.02	1.25 b	0.17
2003	AB	DL	4.22 b	0.21	0.97 a	0.42	0.99 b	0.13	0.28 b	0.19	0.06 a	0.00	0.45 a	0.26
2003	AB	L	4.04 ab	0.08	0.80 a	0.05	0.85 b	0.15	0.06 a	0.04	0.06 a	0.01	1.06 b	0.48
2004	Ap1	C	3.84 a	0.05	2.40 b	0.33	0.59 a	0.30	0.02 a	0.01	0.08 a	0.04	3.01 b	0.45
2004	Ap1	DL	6.74 c	0.52	2.30 ab	0.11	4.19 b	1.47	1.22 b	0.83	0.05 a	0.01	1.01 a	0.47
2004	Ap1	L	5.32 b	0.65	2.01 a	0.15	3.70 b	0.28	0.09 a	0.05	0.05 a	0.01	0.47 a	0.11
2004	Ap2	C	4.01 a	0.25	2.20 a	0.94	0.30 a	0.20	0.02 a	0.01	0.08 b	0.04	1.77 b	0.18
2004	Ap2	DL	5.01 b	0.27	1.83 a	0.52	1.60 b	0.03	1.36 b	0.95	0.04 ab	0.01	1.57 ab	0.67
2004	Ap2	L	4.36 a	0.24	2.34 a	0.11	1.65 b	0.20	0.06 a	0.03	0.06 a	0.01	0.84 a	0.66
2004	AB	C	4.00 a	0.08	0.67 a	0.11	0.53 a	0.43	0.05 a	0.04	0.04 a	0.01	2.17 a	1.20
2004	AB	DL	4.15 a	0.18	0.83 a	0.09	0.76 a	0.36	0.54 b	0.45	0.04 a	0.01	2.05 a	0.86
2004	AB	L	4.11 a	0.06	0.79 a	0.18	0.98 a	0.13	0.08 a	0.06	0.04 a	0.01	1.96 a	0.65
2005	Ap1	C	3.74 a	0.06	2.33 a	0.08	0.07 a	0.04	0.02 a	0.01	0.14 b	0.05	1.59 b	0.37
2005	Ap1	DL	6.42 c	0.23	2.37 a	0.76	7.02 b	1.83	1.53 b	0.53	0.09 a	0.03	0.88 a	0.70
2005	Ap1	L	5.26 b	0.24	2.51 a	0.41	5.25 b	1.51	0.11 a	0.01	0.11 ab	0.02	0.54 a	0.23
2005	Ap2	C	3.76 a	0.04	2.30 a	0.23	0.21 a	0.13	0.03 a	0.01	0.10 b	0.04	2.13 b	0.43
2005	Ap2	DL	4.34 c	0.24	2.43 a	0.66	1.40 c	0.42	1.16 b	0.23	0.05 a	0.00	0.56 a	0.35
2005	Ap2	L	4.05 b	0.14	2.03 a	0.13	0.91 b	0.11	0.02 a	0.01	0.04 a	0.02	1.67 b	0.41
2005	AB	C	3.84 a	0.03	0.65 a	0.22	0.16 a	0.06	0.01 a	0.01	0.03 a	0.03	2.04 a	0.53
2005	AB	DL	3.93 a	0.19	1.03 a	0.50	0.36 ab	0.20	0.47 b	0.12	0.04 a	0.02	1.70 a	0.52
2005	AB	L	3.88 a	0.04	0.81 a	0.15	0.61 b	0.29	0.04 a	0.04	0.03 a	0.02	2.31 a	0.29

Y	D	T	pH		SOM		Ca		Mg		K		Al	
			**Mean**	**SD**	**Mean**	**SD**	**Mean**	**SD**	**Mean**	**SD**	**Mean**	**SD**	**Mean**	**SD**
2006	Ap1	C	3.93 a	0.09	2.54 a	0.44	0.24 a	0.15	0.02 a	0.02	0.11 b	0.04	2.04 b	0.92
2006	Ap1	DL	5.77 b	1.23	2.24 a	0.42	5.98 b	2.18	0.90 b	0.32	0.08 ab	0.03	0.46 a	0.22
2006	Ap1	L	5.49 b	0.35	2.15 a	0.47	4.72 b	1.37	0.05 a	0.02	0.07 a	0.02	0.35 a	0.25
2006	Ap2	C	4.07 a	0.14	1.72 a	0.38	0.08 a	0.03	0.02 a	0.00	0.08 b	0.04	1.37 b	0.24
2006	Ap2	DL	5.06 b	0.86	1.67 a	0.25	2.83 b	2.42	1.23 b	0.35	0.06 ab	0.04	0.36 a	0.15
2006	Ap2	L	4.15 a	0.06	2.23 b	0.26	1.26 ab	0.03	0.01 a	0.01	0.01 a	0.01	1.59 b	0.89
2006	AB	C	3.92 a	0.06	0.48 a	0.04	0.41 a	0.30	0.04 a	0.03	0.02 a	0.02	1.51 a	0.43
2006	AB	DL	3.95 a	0.08	0.57 a	0.10	0.65 a	0.37	0.80 b	0.30	0.03 a	0.02	1.57 a	0.78
2006	AB	L	4.00 a	0.12	1.02 b	0.28	0.77 a	0.51	0.02 a	0.02	0.01 a	0.01	3.20 b	0.53
2007	Ap1	C	3.92 a	0.06	2.22 a	0.31	0.27 a	0.17	0.08 a	0.03	0.08 b	0.03	2.90 b	1.31
2007	Ap1	DL	6.04 c	0.66	2.43 a	0.65	3.71 b	0.92	0.97 b	0.39	0.08 b	0.01	0.63 a	0.32
2007	Ap1	L	4.67 b	0.27	2.58 a	0.32	2.60 b	1.00	0.04 a	0.03	0.05 a	0.01	0.57 a	0.29
2007	Ap2	C	3.98 a	0.05	1.92 a	0.46	0.18 a	0.17	0.09 a	0.02	0.06 a	0.02	3.06 b	1.24
2007	Ap2	DL	4.57 c	0.07	1.97 a	0.27	1.03 b	0.11	0.95 b	0.29	0.05 a	0.03	0.93 a	0.38
2007	Ap2	L	4.30 b	0.17	2.18 a	0.31	1.57 c	0.45	0.03 a	0.03	0.03 a	0.02	1.04 a	0.42
2007	AB	C	4.05 a	0.06	0.58 a	0.18	0.13 a	0.09	0.08 a	0.03	0.06 b	0.02	2.92 a	1.24
2007	AB	DL	4.00 a	0.16	0.63 a	0.09	0.26 a	0.22	0.19 b	0.06	0.02 a	0.01	2.58 a	0.78
2007	AB	L	4.00 a	0.05	0.66 a	0.12	0.46 b	0.06	0.07 a	0.01	0.02 a	0.01	2.17 a	0.54
2008	Ap1	C	4.07 a	0.11	2.12 a	0.15	0.09 a	0.08	0.05 a	0.01	0.09 a	0.04	2.10 b	0.92
2008	Ap1	DL	6.07 c	0.38	2.13 a	0.13	3.78 b	1.20	0.88 b	0.07	0.07 a	0.04	1.04 ab	0.70
2008	Ap1	L	5.48 b	0.36	2.29 a	0.13	3.65 b	0.63	0.09 a	0.02	0.07 a	0.06	0.82 a	0.61
2008	Ap2	C	4.11 a	0.04	2.07 a	0.48	0.10 a	0.08	0.04 a	0.01	0.09 a	0.03	1.49 b	0.34
2008	Ap2	DL	5.56 b	0.75	1.99 a	0.39	2.97 b	0.87	0.74 b	0.26	0.12 a	0.10	0.51 a	0.10
2008	Ap2	L	4.72 a	0.25	2.31 a	0.29	2.33 b	0.16	0.04 a	0.02	0.05 a	0.03	0.59 a	0.25
2008	AB	C	4.16 a	0.15	0.89 a	0.14	0.16 a	0.07	0.04 a	0.02	0.06 b	0.01	2.16 b	0.85
2008	AB	DL	4.98 b	0.27	1.44 ab	0.66	1.72 b	0.27	0.97 b	0.23	0.04 a	0.01	0.99 a	0.37
2008	AB	L	4.42 a	0.23	1.67 b	0.14	1.52 b	0.78	0.03 a	0.02	0.03 a	0.03	1.41 ab	0.89
2009	Ap1	C	3.98 a	0.33	1.54 a	0.41	0.16 a	0.12	0.05 a	0.02	0.10 b	0.03	2.72 c	0.16
2009	Ap1	DL	5.46 b	0.82	1.57 a	0.46	3.58 c	0.42	0.84 b	0.66	0.07 ab	0.05	0.97 a	0.16
2009	Ap1	L	4.53 a	0.04	2.03 a	0.14	1.98 b	0.14	0.07 a	0.03	0.04 a	0.00	1.44 b	0.28
2009	Ap2	C	3.94 a	0.09	1.57 a	0.11	0.22 a	0.16	0.04 a	0.02	0.07 b	0.02	2.05 b	0.20
2009	Ap2	DL	4.60 b	0.27	1.62 a	0.30	1.56 b	0.44	0.78 b	0.16	0.03 a	0.01	0.84 a	0.46
2009	Ap2	L	4.15 a	0.17	1.76 a	0.18	1.12 b	0.61	0.05 a	0.03	0.02 a	0.01	1.68 ab	1.26
2009	AB	C	4.05 a	0.02	0.67 a	0.20	0.23 a	0.17	0.04 a	0.02	0.02 a	0.02	2.54 a	1.03
2009	AB	DL	4.19 b	0.04	0.62 a	0.19	0.58 b	0.22	0.45 b	0.35	0.01 a	0.01	1.62 a	0.53
2009	AB	L	4.04 a	0.06	0.68 a	0.11	0.39 ab	0.15	0.04 a	0.01	0.01 a	0.01	2.19 a	0.50
2010	Ap1	C	3.83 a	0.09	2.03 a	0.41	0.14 a	0.06	0.05 a	0.04	0.12 a	0.03	2.31 b	0.52
2010	Ap1	DL	5.62 c	0.02	1.85 a	0.03	3.64 b	0.63	1.03 b	0.49	0.09 a	0.01	0.20 a	0.08
2010	Ap1	L	4.81 b	0.36	1.90 a	0.03	2.87 b	0.88	0.03 a	0.02	0.10 a	0.04	0.25 a	0.01
2010	Ap2	C	4.12 a	0.38	1.77 a	0.31	0.11 a	0.06	0.06 a	0.03	0.11 b	0.02	1.62 b	0.56

Y	D	T	pH		SOM		Ca		Mg		K		Al	
			**Mean**	**SD**	**Mean**	**SD**	**Mean**	**SD**	**Mean**	**SD**	**Mean**	**SD**	**Mean**	**SD**
2010	Ap2	DL	4.70 a	0.57	1.58 a	0.13	1.63 b	0.44	0.67 b	0.17	0.07 a	0.00	0.80 a	0.56
2010	Ap2	L	4.42 a	0.31	1.76 a	0.09	1.96 b	0.85	0.06 a	0.03	0.09 ab	0.03	0.68 a	0.52
2010	AB	C	4.31 a	0.35	0.70 a	0.13	0.12 a	0.10	0.06 a	0.01	0.09 b	0.03	2.62 a	0.98
2010	AB	DL	4.08 a	0.09	0.60 a	0.09	0.40 a	0.12	0.24 b	0.12	0.06 a	0.01	2.33 a	1.23
2010	AB	L	4.04 a	0.12	0.63 a	0.16	0.91 b	0.42	0.05 a	0.04	0.08 ab	0.01	2.32 a	1.37
2011	Ap1	C	3.99 a	0.08	2.02 a	0.10	0.23 a	0.03	0.15 a	0.02	0.25 a	0.09	1.84 b	0.52
2011	Ap1	DL	5.00 b	0.27	2.20 a	0.45	4.31 b	1.31	1.27 b	0.37	0.23 a	0.14	0.56 a	0.31
2011	Ap1	L	4.77 b	0.13	2.12 a	0.10	4.00 b	0.63	0.26 a	0.02	0.43 a	0.28	1.07 a	0.41
2011	Ap2	C	4.06 a	0.11	1.51 a	0.53	0.09 a	0.04	0.14 a	0.01	0.24 a	0.09	2.52 a	0.36
2011	Ap2	DL	4.94 b	0.42	1.95 a	0.86	3.54 c	1.02	1.20 b	0.25	0.23 a	0.13	1.76 a	1.77
2011	Ap2	L	4.38 a	0.12	2.14 a	0.20	2.36 b	0.30	0.21 a	0.02	0.33 a	0.18	1.32 a	0.58
2011	AB	C	4.14 a	0.04	0.90 a	0.17	0.09 a	0.04	0.14 a	0.01	0.24 a	0.10	2.16 ab	0.32
2011	AB	DL	4.23 a	0.20	1.77 a	1.10	1.73 b	1.03	0.81 b	0.17	0.23 a	0.14	3.13 b	0.55
2011	AB	L	4.11 a	0.04	1.11 a	0.44	1.13 b	0.39	0.17 a	0.00	0.33 a	0.18	2.06 a	1.04

Table S2. Means and standard deviations (SD) of biomass (Biomass: total rye biomass; Spike: spike rye biomass; Stem: stem rye biomass (all of them in kg/ha)) during ten years (2002-2011). Values within each column followed by different lowercase letters are significantly different according to Tukey's honest significance test with Holm-Bonferroni adjustment (0.05). Y: Year of sampling; T: Liming treatment (C: control; DL: dolomitic limestone; L: limestone).

Y	T	Biomass		Spike		Stem	
		Mean	SD	Mean	SD	Mean	SD
2002	C	2740 a	116	1190 a	79.3	1550 a	41.7
2002	DL	5230 b	513	2260 c	181	2970 b	340
2002	L	4770 b	467	2030 b	148	2740 b	322
2003	C	2100 a	228	836 a	62.4	1270 a	166
2003	DL	3450 b	501	1430 b	258	2030 b	243
2003	L	3440 b	117	1340 b	73.0	2100 b	76.0
2004	C	1210 a	177	515 a	142	693 a	42.8
2004	DL	2970 c	295	1310 c	110	1670 c	187
2004	L	2360 b	404	1000 b	193	1350 b	217
2005	C	1580 a	185	578 a	27.9	997 a	159
2005	DL	2820 b	203	1180 b	42.7	1640 b	203
2005	L	2920 b	116	1210 b	36.4	1720 b	104
2006	C	2850 a	340	1350 a	294	1500 a	58.6
2006	DL	3970 b	105	1860 b	63.0	2110 b	45.4
2006	L	4300 b	240	1990 b	85.1	2320 c	171
2007	C	173 a	74.7	80.0 a	35.5	93.4 a	39.3
2007	DL	1000 b	200	431 b	96.7	565 b	121
2007	L	1090 b	187	452 b	116	633 b	77.5
2008	C	2430 a	365	968 a	151	1470 a	220
2008	DL	2430 a	1250	1000 a	488	1430 a	766
2008	L	2910 a	681	1220 a	280	1690 a	403
2009	C	1760 a	45.5	842 a	42.7	915 a	57.1
2009	DL	2990 b	411	1470 b	217	1510 b	193
2009	L	2830 b	433	1410 b	208	1430 b	233
2010	C	3030 a	72.7	1300 a	20.9	1720 a	58.2
2010	DL	3850 b	294	1710 b	133	2140 b	186
2010	L	3550 b	317	1610 b	188	1950 b	138
2011	C	2440 a	279	1070 a	106	1360 a	173
2011	DL	3580 b	375	1520 b	38.4	2060 b	346
2011	L	3210 b	400	1430 b	140	1780 b	267

Table S3. Means and standard deviations (SD) of calcium, magnesium and potassium content in rye biomass (Ca-Rye, Mg-Rye and K-Rye respectively; all of them in %) during ten years (2002-2011). Values within each column followed by different lowercase letters are significantly different according to Tukey's honest significance test with Holm-Bonferroni adjustment (0.05). Y: Year of sampling; T: Liming treatment (C: control; DL: dolomitic limestone; L: limestone).

Y	T	Ca-Rye		Mg-Rye		K-Rye	
		Mean	SD	Mean	SD	Mean	SD
2002	C	0.10 a	0.04	0.04 a	0.01	0.73 b	0.09
2002	DL	0.11 a	0.02	0.18 b	0.06	0.48 a	0.10
2002	L	0.15 a	0.04	0.05 a	0.01	0.59 ab	0.15
2003	C	0.17 b	0.02	0.03 a	0.01	0.29 a	0.05
2003	DL	0.13 a	0.03	0.12 b	0.03	0.42 b	0.02
2003	L	0.20 b	0.03	0.04 a	0.00	0.37 b	0.02
2004	C	0.13 a	0.03	0.02 a	0.01	0.54 a	0.03
2004	DL	0.14 a	0.03	0.13 b	0.03	0.55 a	0.04
2004	L	0.22 b	0.04	0.03 a	0.00	0.47 a	0.08
2005	C	0.14 a	0.06	0.03 a	0.00	0.79 a	0.21
2005	DL	0.15 a	0.05	0.12 b	0.04	0.63 a	0.01
2005	L	0.22 b	0.03	0.03 a	0.01	0.73 a	0.08
2006	C	0.08 a	0.01	0.02 a	0.01	0.46 b	0.01
2006	DL	0.09 a	0.03	0.08 b	0.02	0.38 a	0.08
2006	L	0.14 b	0.04	0.03 a	0.00	0.45 ab	0.04
2007	C	0.07 a	0.01	0.02 a	0.01	0.48 a	0.08
2007	DL	0.20 ab	0.13	0.11 c	0.02	0.52 a	0.17
2007	L	0.25 b	0.11	0.04 b	0.00	0.58 a	0.13
2008	C	0.12 a	0.02	0.02 a	0.00	0.65 a	0.04
2008	DL	0.17 b	0.04	0.09 c	0.02	0.73 a	0.22
2008	L	0.20 b	0.01	0.04 b	0.01	0.67 a	0.04
2009	C	0.10 a	0.01	0.02 a	0.01	0.46 b	0.05
2009	DL	0.19 b	0.01	0.10 c	0.01	0.37 a	0.02
2009	L	0.20 b	0.02	0.04 b	0.01	0.35 a	0.03
2010	C	0.12 a	0.00	0.02 a	0.01	0.45 a	0.07
2010	DL	0.18 b	0.04	0.11 c	0.00	0.39 a	0.10
2010	L	0.24 c	0.03	0.05 b	0.02	0.45 a	0.08
2011	C	0.11 a	0.04	0.02 a	0.01	0.44 a	0.07
2011	DL	0.13 a	0.06	0.07 c	0.02	0.44 a	0.10
2011	L	0.08 a	0.02	0.04 b	0.00	0.39 a	0.13
